# The relationship between ethnicity and multiple sclerosis characteristics in the United Kingdom: A UK MS Register study

**DOI:** 10.1177/13524585241277018

**Published:** 2024-09-20

**Authors:** Benjamin M Jacobs, Luisa Schalk, Emily Tregaskis-Daniels, Pooja Tank, Sadid Hoque, Michelle Peter, Katherine Tuite-Dalton, James Witts, UK MS Register Study Group, Riley Bove, Ruth Dobson

**Affiliations:** Centre for Preventive Neurology, Wolfson Institute of Population Health, Queen Mary University of London, London, UK Department of Neurology, Royal London Hospital, London, UK; Centre for Preventive Neurology, Wolfson Institute of Population Health, Queen Mary University of London, UK; Centre for Preventive Neurology, Wolfson Institute of Population Health, Queen Mary University of London, UK; Centre for Preventive Neurology, Wolfson Institute of Population Health, Queen Mary University of London, UK; Centre for Preventive Neurology, Wolfson Institute of Population Health, Queen Mary University of London, UK; Centre for Preventive Neurology, Wolfson Institute of Population Health, Queen Mary University of London, UK; UK MS Register, University of Swansea, Swansea, UK; UK MS Register, University of Swansea, Swansea, UK; UCSF Weill Institute for Neurosciences, UCSF, San Francisco, CA, USA; Centre for Preventive Neurology, Wolfson Institute of Population Health, Queen Mary University of London, London, UK Department of Neurology, Royal London Hospital, London, UK

**Keywords:** Ethnicity, multiple sclerosis, severity, healthcare inequality

## Abstract

**Background:**

Previous studies have suggested differences in multiple sclerosis (MS) severity according to ethnicity.

**Methods:**

Data were obtained from the UK MS Register, a prospective longitudinal cohort study of persons with MS. We examined the association between self-reported ethnic background and age at onset, symptom of onset and a variety of participant-reported severity measures. We used adjusted multivariable linear regression models to explore the association between ethnicity and impact of MS, and Cox proportional hazards models to assess disability progression.

**Results:**

We analysed data from 17,314 people with MS, including participants from self-reported Black (*n* = 157) or South Asian (*n* = 230) ethnic backgrounds. Age at MS onset and diagnosis was lower in those of South Asian (median 30.0) and Black (median 33.0) ethnicity compared with White ethnicity (median 35.0). In participants with online MS severity measures available, we found no statistically significant evidence for an association between ethnic background and physical disability in MS in both cross-sectional and longitudinal analyses.

**Conclusion:**

We found no association between ethnic background and MS severity in a large, diverse UK cohort. These findings suggest that other factors, such as socioeconomic status and structural inequalities, may explain previous findings.

## Introduction

Multiple sclerosis (MS) affects individuals from all ethnic backgrounds.^1–9^ It has been suggested that ethnicity may be associated with differences in MS disease course, including age at onset, speed of progression and response to treatment. Specifically, previous studies have suggested that MS has a more aggressive course in people from Black and Hispanic ethnic backgrounds, with earlier onset, more rapid disability progression, greater cognitive and physical impairment, and poorer response to disease-modifying therapies (DMTs).^10–21^ Other studies have shown an earlier age at onset in Southeast Asian^22^ and South Asian persons.^23,24^

It remains unclear whether these associations reflect a biological correlate of ethnicity – such as genetic ancestry – or whether the observed differences are driven by social determinants of health, such as systemic or medical racism, healthcare inequalities and poorer access to timely diagnosis and treatment among minority ethnic groups.^25^ Clarifying whether ethnicity is itself associated with an unfavourable MS prognosis is crucial to further exploration of factors responsible for disease progression.

Answering this question robustly requires prospective data from population-based cohorts which are diverse in terms of ethnicity, geographic location and healthcare provider. There have been no studies examining the relationship between ethnicity and MS severity in large, longitudinal cohorts of this nature to date; and specifically, none in countries where universal access to healthcare is routine. The UK MS Register (UKMSR) is a longitudinal cohort study of adults with MS living in the United Kingdom, which has recruited more than 20,000 people with MS since 2011.^26,27^ This resource offers a unique opportunity to study potential contributors to disease severity in a longitudinal cohort. The UKMSR collects a range of participant-reported outcome measures (PROMs) which provide insight into multiple dimensions of disability in MS, including fatigue, mood, and quality of life in addition to traditional scores based largely on physical disability i.e. the online version of the Expanded Disability Status Scale (EDSS).

In this paper, we use the UKMSR to explore the association between self-reported ethnic background and MS severity measures across a range of domains. We use both patient-reported and disease-specific disability measures to probe the impact of MS severity across different ethnic groups, examining both cross-sectional estimates and longitudinal trajectories of severity and impact.

## Methods

### Cohort

Data were extracted from the UKMSR in November 2023. Details of participant recruitment and study design have been previously described.^26,27^ In brief, baseline demographic characteristics (including age at onset, recruitment and self-stated gender), self-reported disease type, treatment details and lifestyle information are gathered at recruitment, and participants are surveyed on a 6-month basis using a battery of PROMs (Table 1, **Supplementary** Figure 1).

**Table 1.** Characteristics of the cohort.

	White	Black	South Asian	*p*
	*N* = 16,927	*N* = 157	*N* = 230	
Age at diagnosis	38.0 [31.0, 46.0]	36.0 [29.0, 44.0]	32.0 [26.0, 39.0]	<0.001
Year of diagnosis	2009 [2001, 2015]	2012 [2008, 2016]	2013 [2009, 2017]	<0.001
Gender:				0.001
Female	12,690 (75.0%)	115 (73.2%)	147 (63.9%)	
Male	4237 (25.0%)	42 (26.8%)	83 (36.1%)	
University education:				<0.001
No	8785 (57.9%)	71 (51.8%)	55 (26.7%)	
Yes	6397 (42.1%)	66 (48.2%)	151 (73.3%)	
Age at symptom onset	34.0 [27.0, 42.0]	33.0 [27.0, 41.0]	30.0 [24.0, 36.0]	<0.001
DMT prior to baseline:				0.059
High efficacy	784 (4.63%)	5 (3.18%)	18 (7.83%)	
Low efficacy	1024 (6.05%)	6 (3.82%)	18 (7.83%)	
No DMT data available	15,119 (89.3%)	146 (93.0%)	194 (84.3%)	
PPMS:				0.97
No	14,706 (86.9%)	136 (86.6%)	201 (87.4%)	
Yes	2221 (13.1%)	21 (13.4%)	29 (12.6%)	
Baseline EDSS	5.00 [3.00, 6.50]	4.50 [3.00, 6.50]	4.00 [2.00, 6.50]	0.002
Age at baseline EDSS	52.4 [43.5, 59.9]	48.4 [36.5, 54.7]	43.2 [35.2, 50.7]	<0.001
Baseline gARMSS	6.33 [4.24, 8.00]	6.48 [4.89, 8.30]	6.52 [4.28, 8.33]	0.213
Baseline MSIS	41.7 [20.0, 63.3]	35.0 [15.0, 56.7]	35.0 [16.2, 62.1]	0.109
Age at baseline MSIS	49.8 [41.2, 57.7]	45.6 [36.5, 51.8]	40.5 [34.2, 46.8]	<0.001

‘High-efficacy DMT’ refers to any of alemtuzumab, cladribine, fingolimod, natalizumab, ocrelizumab, siponimod and ofatumumab. ‘Low-efficacy DMT’ refers to any of beta-interferon, dimethyl fumarate, glatiramer acetate and teriflunomide.

### Variable definitions

#### Demographic variables and ethnicity

Self-reported ethnic background is selected by participants at registration using the UK 2001 Census categories. Granular categories were condensed into four parent groups (White, Asian, Black, Mixed/other). Other demographic variables obtained via self-report include age at MS symptom onset, age at diagnosis, MS type at onset, DMT exposure, educational attainment and gender. DMT exposure was defined using self-reported DMT records indicating the drug had started prior to study enrolment. We defined three categories of DMT exposure: those with no data, those exposed to low-efficacy DMT (interferon, glatiramer acetate, dimethyl fumarate, teriflunomide) and those exposed to high-efficacy DMT (natalizumab, anti-CD20 therapy (ocrelizumab, ofatumumab), S1P inhibitors (fingolimod, siponimod), cladribine and alemtuzumab).

#### MS outcome measures

We used five separate outcome measures of MS severity: the online (web) EDSS and four PROMs: the physical dimension of the MS Impact Scale (MSIS29 version 2), the EuroQol 5D-3L/5L Visual Analogue Scale (VAS), the Fatigue Severity Scale (FSS) and the MS Walking Scale (MSWS). From this point, we refer to the normalised physical MSIS score, the normalised FSS and the normalised MSWS as the MSIS, FSS and MSWS, respectively (**Supplementary** Figure 2).

## Cohort definition

### Primary analysis (cross-sectional) cohort

**Figure 1.**
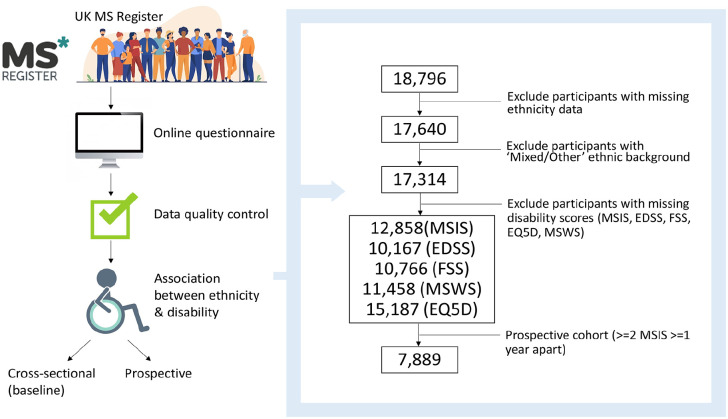
Flowchart depicting the experiment design, including participant inclusion/exclusion criteria.

From an initial 21,643 participants with non-missing gender and a valid age at diagnosis (⩾ 18 and ⩽ 90), we removed individuals with missing data for the following parameters, with the resulting sample size given in the brackets (Figure 1):

*N* = 1112 with missing age at symptom onset (*N* = 20,531);
*N* = 1735 with missing MS subtype at diagnosis (*N* = 18,796);
*N* = 1156 with missing ethnicity (*N* = 17,640);
*N* = 326 with ‘Other’ ethnicity (*N* = 17,314).

These exclusions resulted in a dataset of 17,314 participants enrolled in the UKMSR with complete demographic data (age, sex, MS type at onset, age at MS symptom onset and diagnosis, ethnicity and year of birth; Table 1; Figure 1) and who identified as one of the following ethnic groups: ‘I am Asian or British Asian (Indian / Pakistani / Bangladeshi’, ‘I am Black or Black British (Caribbean, African, Other)’, ‘I am white (British, Irish, Other)’, referred to from this point onwards as ‘South Asian’, ‘Black’, ‘White’, respectively. We defined a matched cohort by matching each Black and South Asian person with MS to exactly two White participants with identical self-stated gender, year of diagnosis (rounded to the nearest year), age at diagnosis (rounded to the nearest year) and MS subtype at diagnosis (relapsing vs progressive onset).

### Longitudinal cohort

To assess the relationship between ethnicity and long-term disability progression in a longitudinal setting, we defined a longitudinal cohort by identifying participants from the cross-sectional cohort who met the following criteria:

1. At least two valid MSIS recordings – the earliest (baseline) reading and at least one reading within 5 years of the baseline.
2. Baseline MSIS ⩽ 90.
3. Follow-up reading ⩾ 1 year from the baseline

### Statistical analysis

#### Comparison of demographic characteristics

To compare the demographic baseline characteristics between groups, we used Kruskal–Wallis tests (for continuous variables) and chi-square tests (for categorical variables). Unless specified, categorical variables are reported as *n* (%) and continuous variables as median (interquartile range).

#### Association of ethnicity with clinical characteristics

We tested for association between ethnicity and age at MS onset and diagnosis using linear regression models. Models were adjusted for gender and self-reported progressive onset disease. Visual inspection of the distributions of both age at symptom onset and age at diagnosis confirmed both were normally distributed. Inspection of models revealed that linear regression assumptions of linearity and normally distributed variances were satisfied. We derived an estimated time from onset to diagnosis as the difference between the stated age at onset and age at diagnosis. As this variable was not normally distributed, we tested for association with ethnicity using bootstrapped linear regression models adjusted for gender and progressive onset disease. For these models, we excluded participants whose stated the date of symptom onset came after their stated date of diagnosis. We explored the association between ethnicity and onset symptom using multinomial logistic regression models adjusted for gender, progressive onset disease, and age at symptom onset, and calculated confidence intervals and *p*-values from Z-scores.

#### Association of ethnicity with baseline severity outcome measures

Associations between ethnicity and baseline MS severity outcome measures were tested using linear regression models, with the ‘White’ ethnic group as the reference category. For the primary analyses, regression models were adjusted for the age at the time of recording, gender, and self-reported progressive onset disease. The outcome measure (dependent variable) for these regression models was the earliest raw (i.e. untransformed) severity score per participant. Participants who did not have any recorded values for a particular severity score were excluded from the regression model.

The distribution of severity measure scores was not normal, thus violating the assumptions of linear regression. We therefore calculated the confidence intervals, standard errors and *p-*values of regression coefficients with bootstrapping. For each model, we resampled the dataset with replacement and recalculated the regression model with the resampled data. This procedure was repeated 1000 times for each model. 95% confidence intervals were derived from the empirical quantiles of the sampling distribution. As the sampling distribution for the regression coefficients was normally distributed, we calculated two-tailed *p-*values for the null hypothesis that β = 0 by Z-scoring. Regression models were inspected to ensure that the linearity assumption was met. We inspected correlation between predictors to look for evidence of multicollinearity and confirmed the expected correlations between outcomes (**Supplementary** Figure 3).

#### Association of ethnicity with longitudinal severity outcome measures

We examined the relationship between ethnicity and longitudinal disability progression using the MSIS-29 physical subscale. We filtered the cohort to the participants with longitudinal MSIS readings (defined above). For the time-to-event analysis, we defined ‘disability progression’ as the occurrence of an MSIS score 10 points greater than the baseline score, similar to a recent study^24^ using this cohort, as this has been demonstrated to represent a clinically meaningful MSIS increment (which runs from 0 to 100). To distinguish sustained disability progression from transient worsening, which could be a result of relapse-associated worsening, measurement error, or pseudo-relapse, we identified those with a further follow-up MSIS at least 3 months later. Some participants did not have a further follow-up score, and these participants were classified as non-progressors. The time-to-event was defined as the time from the baseline score to the first MSIS score 10 points above baseline. If participants did not experience disability progression during the follow-up period, they were censored, with the censoring time defined as the last (i.e. most recent) MSIS score. We used Cox proportional hazards regression to model the effect of ethnicity of sustained disability progression. In the primary analysis, we adjusted for age at baseline, baseline MSIS, gender and progressive onset disease. Cox models were inspected for linearity of numerical predictors, validity of the proportional hazards assumption and influence of individual observations.

#### Missing data

We performed complete case analysis, that is, for any given model, participants with missing data for either the outcome or any of the covariates were excluded from the model.

### Computing, data and code availability

All analyses were conducted in R version 4.1.3 within the UKMSR secure research environment (UKSERP).^27^ All code used in these analyses is available at https://benjacobs123456.github.io/ukmsr_ethnicity/. Access to UKMSR data is open to all researchers on application. Details of how to apply for the data can be found here: https://ukmsregister.org/Research/WorkingWithUs.

### Ethical approval

The UKMSR has research ethics approval from South West Central Bristol Research Ethics Committee initially as 16/SW/0194 and currently as 21/SW/0085. This project was approved by the UKMSR Scientific Steering Committee.

## Results

### Ethnicity correlates with age of MS onset

The UKMSR cohort (*n* = 17,314 with complete demographic data, Figure 1) was broadly representative of the UK MS population, with a female predominance (*n* = 12,952, 74.8%), primarily relapse onset disease (*n* = 15,043, 86.9%), largely identifying as White (*n* = 16,927, 97.8%)^23^, with median age at diagnosis of 38.0 (IQR 15) and median age at symptom onset of 34.0 (IQR 15). While the gender ratio appeared similar between White (*n* = 12,690, 75.0% female) and Black (*n* = 115, 73.2% female) ethnic groups, the female preponderance was less pronounced among South Asian participants (*n* = 147, 63.9% female). The proportion of participants with self-reported Primary Progressive MS was similar across ethnic groups (Table 1, **Supplementary** Figure 1).

**Figure 2.**
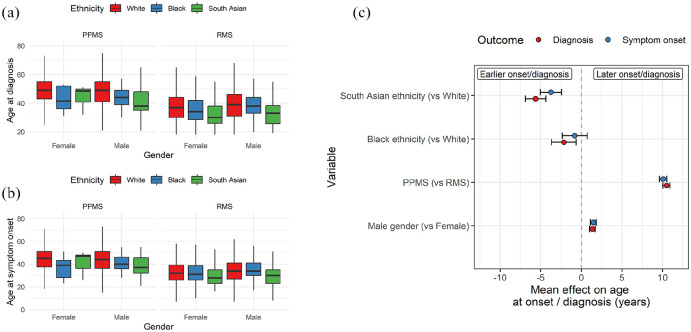
Age at MS onset and diagnosis is younger in South Asian and Black participants. Boxplots showing the age at (a) diagnosis and (b) symptom onset split by ethnicity, MS subtype and gender. (c) Regression coefficients from linear models estimating the impact of ethnicity, MS subtype, and gender on MS onset and age at diagnosis. Points represent the beta coefficient ± 95% CI, that is, the estimated mean difference in age at diagnosis/onset given each variable. The dotted line indicates the null. Red points represent the impact on age at diagnosis, and blue points indicate the impact on age at symptom onset. PPMS – primary progressive MS. RMS – Relapse onset MS.

Participants from Black (*n* = 157) and South Asian (*n* = 230) ethnic backgrounds reported a younger age at both symptom onset and diagnosis than White participants (Figure 2, Table 1, Kruskal–Wallis test, *p* < 0.001). The median age of symptom onset was 30.0 in South Asian participants, 33.0 in Black participants and 34.0 in White participants. The difference in age at diagnosis was even more striking, with South Asian participants diagnosed on average 6 years earlier than White participants (median 32.0 vs 38.0).

The association between Black or South Asian ethnicity and earlier MS diagnosis persisted despite adjustment for gender and progressive onset disease (Black: β = −2.2, *p* = 0.006; South Asian: β = −5.6, *p* = 2.6 × 10^−18^; Figure 2). The associations with symptom onset were consistent in terms of effect direction but had weaker statistical confidence for Black individuals (Black: β = −0.8, *p* = 0.3; South Asian: β = −3.7, *p* = 1.1 × 10^−8^; Figure 2).

These models reproduced the expected finding that male gender and progressive onset disease were associated with later symptom onset and diagnosis. Restricting the analysis to participants diagnosed after 2010, when revisions to the diagnostic criteria made it simpler to diagnose MS based on a single scan (and would therefore plausibly lower the average age at diagnosis) yielded the same findings (e.g. for age at diagnosis, Black: β = −3.5, *p* = 0.002; South Asian: β = −6.3, *p* = 3.5 × 10^−14^).^28^ Similarly, the observation of earlier symptom onset and diagnosis in South Asian and Black participants persisted in a further sensitivity analysis adjusting for year of diagnosis (**Supplementary** Figure 4, e.g. for age at diagnosis, Black: β = −3.3, *p* = 1.1 × 10^−5^; South Asian: β = −7.0, *p* = 1.7 × 10^−29^). Finally, adjusting for year of diagnosis considered as a categorical variable corresponding to the different diagnostic criteria (pre-1983, post-Poser 1983, post-McDonald 2001, post-McDonald 2010 and post-McDonald 2017) yielded the same finding of earlier diagnosis in the South Asian and Black participants, with the effect slightly greater among the South Asian group.

In contrast to recent data from other cohorts, we did not find evidence for delayed diagnosis among participants from minoritised ethnic groups – the lag from symptom onset to diagnosis was less pronounced in Black (median 2.0 years) and South Asian (median 2.0 years) participants compared with White participants (median 3.0 years). As the distribution of lag times was not normally distributed, we adjusted for gender and progressive onset disease using bootstrapped linear regression models. These models confirmed evidence for less diagnostic lag (i.e. shorter time from symptom onset to diagnosis) in the Black (*p* = 0.003) and South Asian (*p* = 0.001) groups. We observed the same finding – of lower diagnostic delay among Black and South Asian participants – in sensitivity analyses either adjusting for year of diagnosis or when restricting to people diagnosed after the 2010 criteria entered practice.

**Figure 3.**
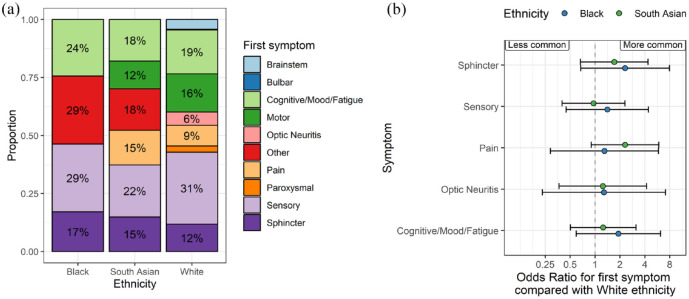
Symptom at MS onset does not differ by ethnicity. (a) Stacked bar plots showing the proportion of people in each ethnic group with the presenting symptom denoted by the colour. Note that the denominator is the number of participants with any reported symptom, which was around one-third of the cohort. Counts < 5 are suppressed and subsumed into the ‘other’ category in the plot. (b) Regression coefficients showing the results of multinomial logistic regression models. In these models, the point estimates refer to the log odds ratio for each symptom compared with motor symptoms at onset, contrasting with the White ethnic group. Beta coefficients and 95% CIs are shown. Symptoms to the right of the null line can be interpreted as ‘more likely to occur as the presenting symptom’ than in the White group, and vice versa for those to the left of the null. None of these associations achieved statistical significance at a *p*-value of less than 0.05. Points are coloured by the ethnic group.

Around one-third of the cohort reported their first MS symptom type (*n* = 4983, 28.8%), including 41 Black, 67 South Asian and 4875 White participants. Overall, sensory symptoms were the most common presenting feature, reported in 1540 participants (30.9% of those with a recorded symptom). Multinomial regression models did not reveal any specific symptom or constellation of symptoms which were associated with ethnic background (at *p* < 0.05), although these estimates were imprecise owing to the small numbers (Figure 3). This result – the lack of statistically significant associations between ethnicity and any one specific symptom – persisted in sensitivity analyses with and without adjustment for age at symptom onset.

### MS severity measures do not differ by ethnicity

**Figure 4.**
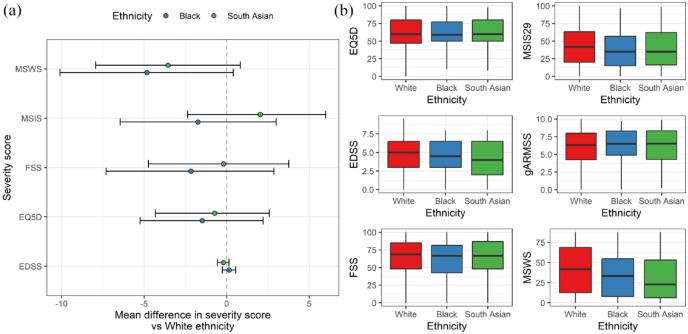
No evidence for association between ethnicity and baseline MS severity measures. (a) Forest plot showing the regression coefficients and 95% CIs from bootstrapped linear regression models examining the impact of ethnic group on baseline severity scores. Note that all scores except for EQ5D are orientated such that higher value equates to ‘worse’ disease, whereas for EQ5D, higher scores indicate higher quality of life. Also note that these values are on the original scale, that is, the EDSS scale runs from 0 to 10, whereas the other scores run from 0 to 100. (b) Boxplots showing the raw distributions of these severity scores in each ethnic group. In addition, the global ARMSS score – the age-related EDSS – is also shown.

The baseline age-adjusted EDSS scores (gARMSS) were similar across ethnic groups (*p* = 0.2, medians 6.25, 6.5 and 6.5 in the White, South Asian and Black groups, respectively, Table 1). In models adjusted for age, gender and progressive onset disease, there was no evidence (*p* < 0.05) for an association between Black or South Asian ethnicity and any of the MS severity scores we studied (Figure 4). Empirical power calculations suggested that we would have > 90% power to detect a clinically meaningful difference of > 10 on the normalised MSIS scale between groups for both the Black and South Asian groups compared with those of White ethnicity. To detect a smaller difference of five points on the normalised MSIS scale, we would have minimal power (42% and 56%, respectively, in the Black and South Asian groups).

We confirmed the expected associations between progressive onset disease, male sex, increasing age and worse measures of MS severity. We also observed a consistent protective effect of both prior DMT exposure and university education, consistent with the known benefits of DMT, and second, the influence of social determinants of health on MS outcomes, as university education is a surrogate for higher socioeconomic status.

We did not find any statistically significant evidence for association between ethnicity and MS severity measures in sensitivity analyses adjusting for age at diagnosis, year of diagnosis, exposure to high-efficacy DMT, educational attainment or age at symptom onset. Sensitivity analysis using the matched cohort in an attempt to further mitigate bias reinforced the previous findings, with no statistically significant relationships between ethnicity and any MS severity score (**Supplementary** Table 1).

### 5-year disability progression is not associated with ethnic background

We defined a longitudinal cohort of 7889 participants (7748 White, 60 Black, 81 South Asian) with at least two MSIS readings separated by at least a year, within 5 years, with the baseline reading ⩽ 90. We identified 3804 participants who had at least one follow-up MSIS greater than 10 points above their baseline score during the 5 years of follow-up. Of these, 2459 (64.6%) had sustained disability progression (i.e. another MSIS greater than 10 points above baseline), 731 (19.2%) had non-sustained progression (i.e. other MSIS scores, but none greater than 10 points above baseline) and 614 (16.1%) did not have a further follow-up score. Participants without a further follow-up score were classified as non-progressors for the primary analysis. The median time to disability progression or censoring of 2.5 years, with a maximum follow-up time of 5 years.

**Figure 5.**
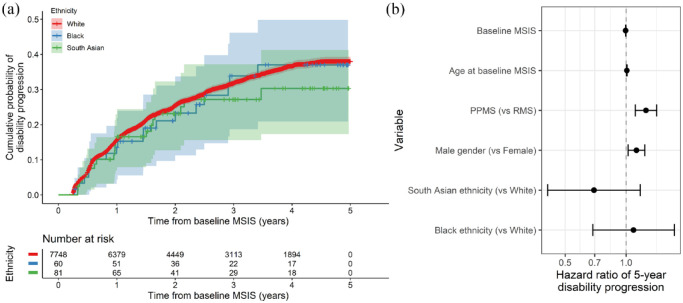
No evidence for association between ethnicity and 5-year disability progression. (a) Survival curves showing the impact of ethnic group on 5-year disability progression as measured by a 10-point increase in the MSIS29 physical subscale. The x-axis indicates time in years from the baseline MSIS measurement, that is, the earliest recorded measurement for each individual. The curves show the cumulative probability of disability progression. Censored individuals are indicated with crosses. However, 95% CIs are shown. (b) Hazard ratios (and 95% CIs) for disability progression given each factor listed on the y-axis. These HRs were derived from a Cox proportional hazards model. PPMS and male gender were associated with higher risk of disability progression, but we did not find any association between ethnic group and progression.

Neither Black nor South Asian ethnicity was associated with sustained disability progression over 5 years of follow-up when compared to those of White ethnicity (HR_Black_ 1.1, 95% CI 0.7–1.7; HR_South Asian_ 0.7, 95% CI 0.4–1.2) (Figure 5a). Factors associated with disability progression were male gender, progressive onset disease, older age at baseline and a lower baseline MSIS score (Figure 5b). We did not find any evidence for a relationship between ethnicity and disability progression across a range of sensitivity analyses, including an unadjusted model, with adjustment for high-efficacy DMT exposure (which showed the expected dose-dependent protective effect of both high-efficacy DMT (HR 0.7, *p* = 0.0004) and low-efficacy DMT (HR 0.81, *p* = 0.005) compared with no DMT / no recorded DMT), with adjustment for age at diagnosis, with adjustment for diagnostic lag (which was itself associated with higher risk of disability progression – HR 1.01, *p* = 0.01), with adjustment for year of diagnosis (which showed the expected association between more recent diagnosis and better outcome – HR 0.99, *p* = 0.003) or in the matched cohort. Empirical power calculations indicated that, given these sample sizes, we would expect > 70% power to detect a 60% increase in the hazard (HR = 1.6) in the Black ethnic group and a 50% increase in the South Asian group (HR = 1.5).

## Discussion

Clarifying whether and how ethnicity impacts on the clinical phenotype of MS is important for understanding the determinants of disability, providing accurate prognostic information and identifying possible healthcare disparities, such as unequal access to timely diagnosis, support and highly effective DMTs. We used data from a large longitudinal cohort study including 387 individuals from Black and South Asian ethnic backgrounds, and showed that these individuals tend to be diagnosed with MS at an earlier age, but do not appear to have a more severe disease course. We did not find evidence of a relationship between ethnicity and severity across a range of participant-reported outcomes ascertained at baseline, or in a longitudinal analysis of sustained disability progression.

Our finding that age at MS onset is earlier in people of Black and South Asian ethnic groups is consistent with our recent UK population-based study from the Clinical Practice Research Datalink (CPRD),^23^ with baseline phenotype data from our genetic cohort study of ancestrally diverse people with MS,^29^ and with other UK studies.^8,24,30^ A primary care records study in East London found that age of onset was earlier in people of South Asian ethnicity but not Black ethnicity people compared with White patients.^8^ Data from the United States have been inconsistent regarding the age of onset in Black individuals, with some studies showing earlier onset and some later.^5,31^ Taken together, these findings suggest that people of South Asian ethnicity living in the United Kingdom do experience a younger age of onset, and while this may also be true for people of Black ethnicity living in the United Kingdom, the difference may be less stark.

The lack of association in our study between ethnicity and cross-sectional disability measures or longitudinal disability progression is reassuring in the context of previous data – largely from the United States – showing higher radiological burden, faster progression and decreased time to fixed disability endpoints among Black individuals with MS.^10–21,31^ The available UK data from small cross-sectional studies have suggested higher rates of disability among Black British MS cases, but not among British South Asian cases.^24,32^ Our findings are broadly in line with a recent large-scale analysis of > 50,000 patients from the MSBase cohort,^33^ which found no association between ethnicity and risk of conversion to Secondary Progressive MS. These results may reflect the lack of a true association between ethnicity and disability in MS, insufficient power to detect a subtle effect or cohort-specific characteristics.

An important caveat in interpreting these results is that ethnicity is not in itself a biological concept. It is often used as a crude proxy for genetic ancestry, but in reality, the association between self-reported ethnicity and genetic ancestry is loose. Self-reported ethnicity is an inherently vague concept which incorporates elements of linguistic, religious, cultural, national and other dimensions of identity. In addition, we use broad self-reported ethnicity groups based on the UK Census. Direct comparison of results between different cohorts is therefore extremely challenging – however, studies such as this are useful for understanding the interaction between ethnicity and MS-related outcomes in particular settings. These insights may be specific to the country, healthcare system and population being studied, but when considered together provide vital evidence into potentially modifiable prognostic factors. Social and healthcare inequalities are a major public health concern in both the United Kingdom^34^ and the United States^35^ and may explain a large proportion of the reported differences in MS outcomes between ethnic groups. However, differences in the precise demographics of the UK and the US populations and differences in the healthcare system (insurance-based in the United States; universal access in the United Kingdom) mean that direct comparisons between studies are not particularly helpful. Our results should therefore be carefully contextualised – they represent findings from a voluntary, UK-based cohort with universal access to healthcare who are able to self-monitor via the Internet, and who are engaged with research.

The key strengths of this work are the richness of the phenotyping, which spans multiple PROMs ascertained over time, and the relatively large sample size. The consistent lack of a relationship between ethnicity and severity across scores for different dimensions and domains of MS-related impairment, both visible (walking) and invisible (fatigue), in both cross-sectional and prospective analysis, and in a range of sensitivity analyses accounting for various possible confounders lends confidence to the null result we report.

The major limitation of this study is the risk of bias due to the voluntary nature of participation in the UKMSR. Participation in the UKMSR is influenced by a variety of factors including socioeconomic status, disability, relationship with healthcare professionals and the healthcare system. These factors may, in theory, skew the cohort towards a relatively less disabled population and may mean that we are unable to detect a true association between ethnicity and greater disability. In reality, it appears the bias may operate in the other direction – that is, this population may be more skewed towards a more disabled population with established disease. Regardless of the direction of this bias, relying on voluntary signups rather than population-based recruitment will attract participants who are engaged with healthcare and with research, which will introduce confounding in several domains, including socioeconomic status and access to timely diagnosis and treatment.

Compared with a population-based UK MS cohort, the proportion of Black and South Asian cases was lower in the UKMSR (1.1% Black and 1.6% South Asian in UKMSR vs 2.6% Black and 2.9% South Asian in CPRD)^23^, suggesting a degree of under-representation of diverse ethnic groups and therefore potential bias. However, there is some evidence to suggest that the UKMSR cohort is broadly representative of the MS population in the United Kingdom. First, we observe a wide distribution of EDSS scores, including many individuals at the upper end of the disability scale, suggesting that the distribution has not been artificially curtailed. In addition, it is reassuring that we are able to replicate known factors associated with greater MS severity, such as progressive onset disease and older age. We also aim to mitigate confounding by socioeconomic status by controlling for educational attainment – although an imperfect proxy, this analysis provides some further support for the results we report.

Other limitations of these data include the exclusive reliance on self-reported metrics, the challenge of properly accounting for the effects of DMT, the possibility of residual confounding by factors such as socioeconomic background, and the restriction to only 5 years of follow-up in the longitudinal analysis. While we attempt to mitigate confounding by performing a wide range of sensitivity analyses adjusting for putative confounders, residual confounding is likely. The use of self-reported severity metrics introduces a degree of inevitable ascertainment bias (whereby the most severely affected people are less likely to enter their data when they are unwell), and a degree of subjectivity which is not that case for disability scores assessed by a clinician. However, the fact that we reproduce several well-known associations between prognostic factors (e.g. progressive onset disease, male gender and DMT use) and subsequent disability progression provides confidence in the self-reported severity scores.

Overall, these findings suggest that, while individuals from Black and South Asian ethnic backgrounds tend to develop MS and be diagnosed at a younger age, there is no evidence of an association between ethnic background and MS severity in a large UK longitudinal cohort, the UKMSR. These findings need to be replicated in population-based UK cohorts to mitigate the problem of selection bias. In addition, it would be valuable to complement this kind of quantitative approach with qualitative and social science work examining how the road to a diagnosis and the experience of living with MS may differ between people of different ethnic backgrounds. There remains work to be done to understand why certain cohorts show a strong association between ethnicity and disability, an effect we were unable to replicate. This may relate to factors specific to our cohort or alternatively it may reflect the wider social and healthcare environments.

## Supplemental Material

sj-docx-1-msj-10.1177_13524585241277018 – Supplemental material for The relationship between ethnicity and multiple sclerosis characteristics in the United Kingdom: A UK MS Register study Supplemental material, sj-docx-1-msj-10.1177_13524585241277018 for The relationship between ethnicity and multiple sclerosis characteristics in the United Kingdom: A UK MS Register study by Benjamin M Jacobs, Luisa Schalk, Emily Tregaskis-Daniels, Pooja Tank, Sadid Hoque, Michelle Peter, Katherine Tuite-Dalton, James Witts, Riley Bove and Ruth Dobson in Multiple Sclerosis Journal
