# Supplementary material for: The relationship between ethnicity and multiple sclerosis characteristics in the United Kingdom: A UK MS Register study
Source: Mult Scler. 2024 Sep 20;30(11-12):1544–55. doi: 10.1177/13524585241277018 (PMC11520257; doi:10.1177/13524585241277018)
Supplement: sj-docx-1-msj-10.1177_13524585241277018 – Supplemental material for The relationship between ethnicity and multiple sclerosis characteristics in the United Kingdom: A UK MS Register study [file sj-docx-1-msj-10.1177_13524585241277018.docx]

**Supplementary note:** The relationship between ethnicity and Multiple Sclerosis characteristics in the United Kingdom: a UK MS Register study

**Demographics of the cohort**

After application of the inclusion and exclusion criteria, 17,314 participants had data available for analysis. The histograms below show the demographic characteristics of the cohort prior to exclusion of people with ‘Mixed/Other’ ethnicity.

**
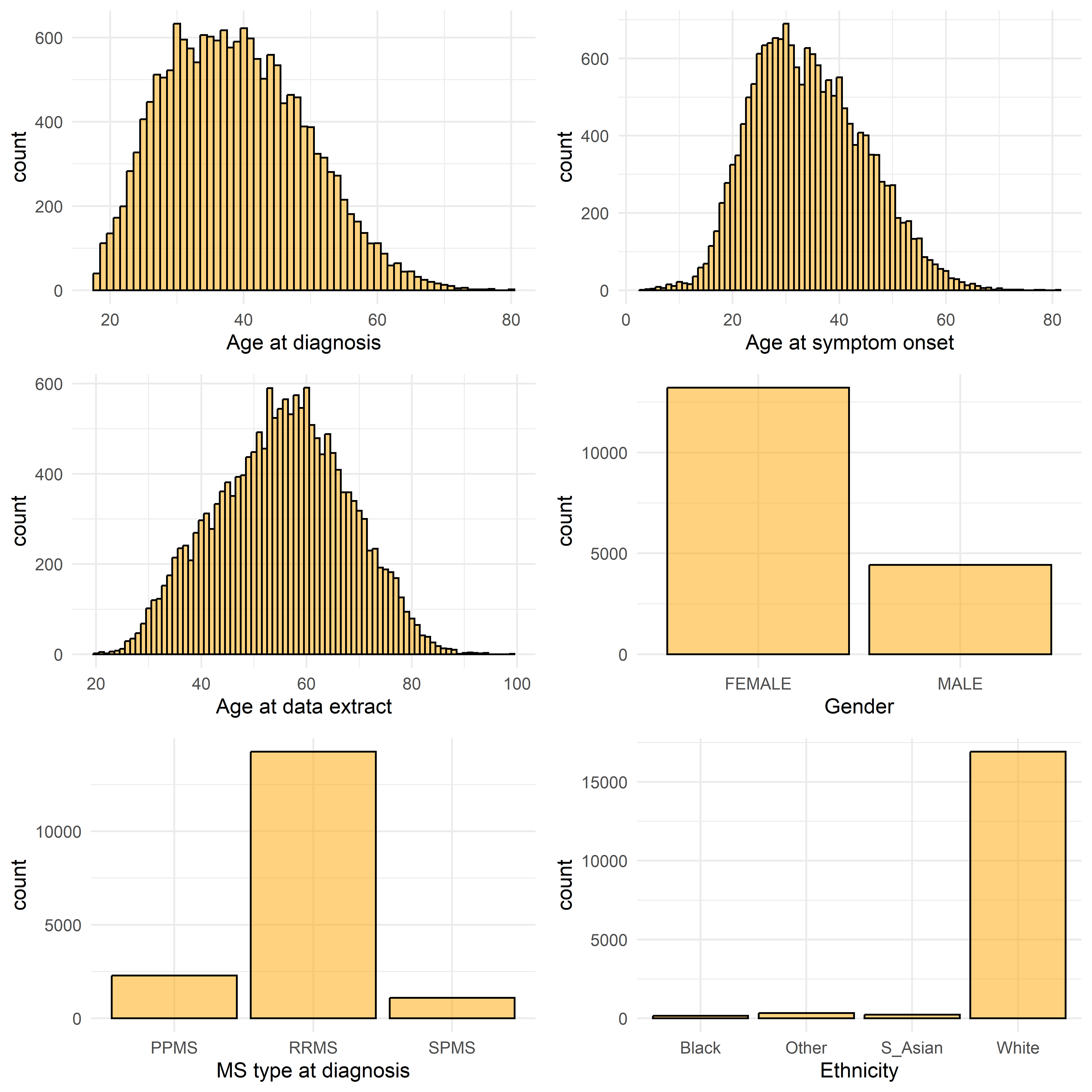
**

Supplementary figure 1: histograms showing the demographic characteristics of the cohorts. Plots show (from left to right and top to bottom) the age at diagnosis, symptom onset, age at data extract, gender, MS subtype at diagnosis, and self-reported ethnic background of the cohort.

**Phenotype data**

EDSS values were evaluated via an online self-administered EDSS scale (the webEDSS^1^). From this we calculated the age-adjusted EDSS score, the ARMSS (global Age-Related Multiple Sclerosis Severity Score; gARMSS) using the ‘ms.sev’ R package^2,3^. The transformation from EDSS to gARMSS accounts for the increase in EDSS with age, allowing meaningful comparison of age-adjusted disability between individuals at different ages:


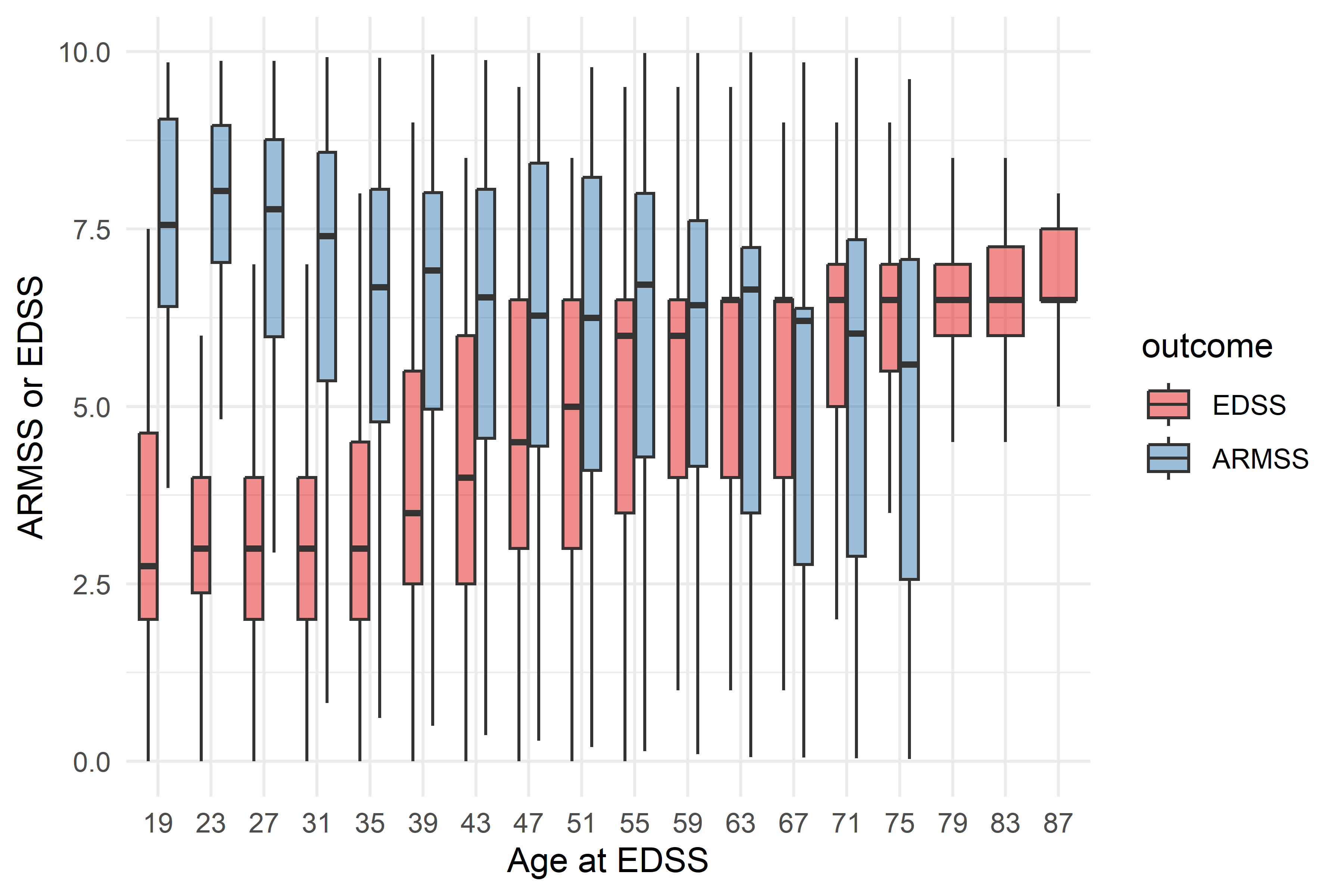


Figure 2: boxplots showing the relationship between age and both EDSS (red boxes) and gARMSS (blue boxes). The gARMSS score reflects an age-adjusted EDSS measure.

We used the 20 ‘physical’ questions of the MSIS29 version 2 to calculate a normalised summary score as previously described^4^. Briefly, each of the questions in the MSIS29-v2 is scored from 1 to 4, with higher scores indicating greater disability. The sum of the 20 questions in the MSIS29 physical domain therefore ranges from 20 to 80. We normalised these scores as follows:

$${Normalised MSIS29}_{Physical}= \frac{{MSIS29}_{Physical}- 20}{60}\times100$$

This procedure yielded normalised MSIS29_Physical_ scores ranging from 0 - 100, with higher scores indicating worse MS. The EQ5D VAS is a self-rating of current health-related quality of life from 0 - 100, with higher scores indicating better quality of life. The fatigue severity score (FSS) consists of seven questions rated from 1 to 9, with 9 indicating a higher degree of impairment due to fatigue. The total score ranges from 9 to 63, with higher scores indicating worse fatigue – these scores were normalised to range from 0 - 100 using the same procedure as for the MSIS^4^,:

$${Normalised FSS}= \frac{FSS- 9}{54}\times100$$

The MS Walking Scale (MSWS) consists of twelve questions evaluating gait which are rated from 1 to 5, with higher scores indicating more severe disability. The raw scores therefore sum to between 12 and 60. Again, these score were normalised to range from 0 - 100 as follows^5^:

$${Normalised MSWS}= \frac{MSWS- 12}{48}\times100$$

We observed the expected correlations between severity measures, with increasing EDSS associated with higher MSIS, greater fatigue (FSS), lower quality of life (EQ5D), and slower walking (MSWS):

_
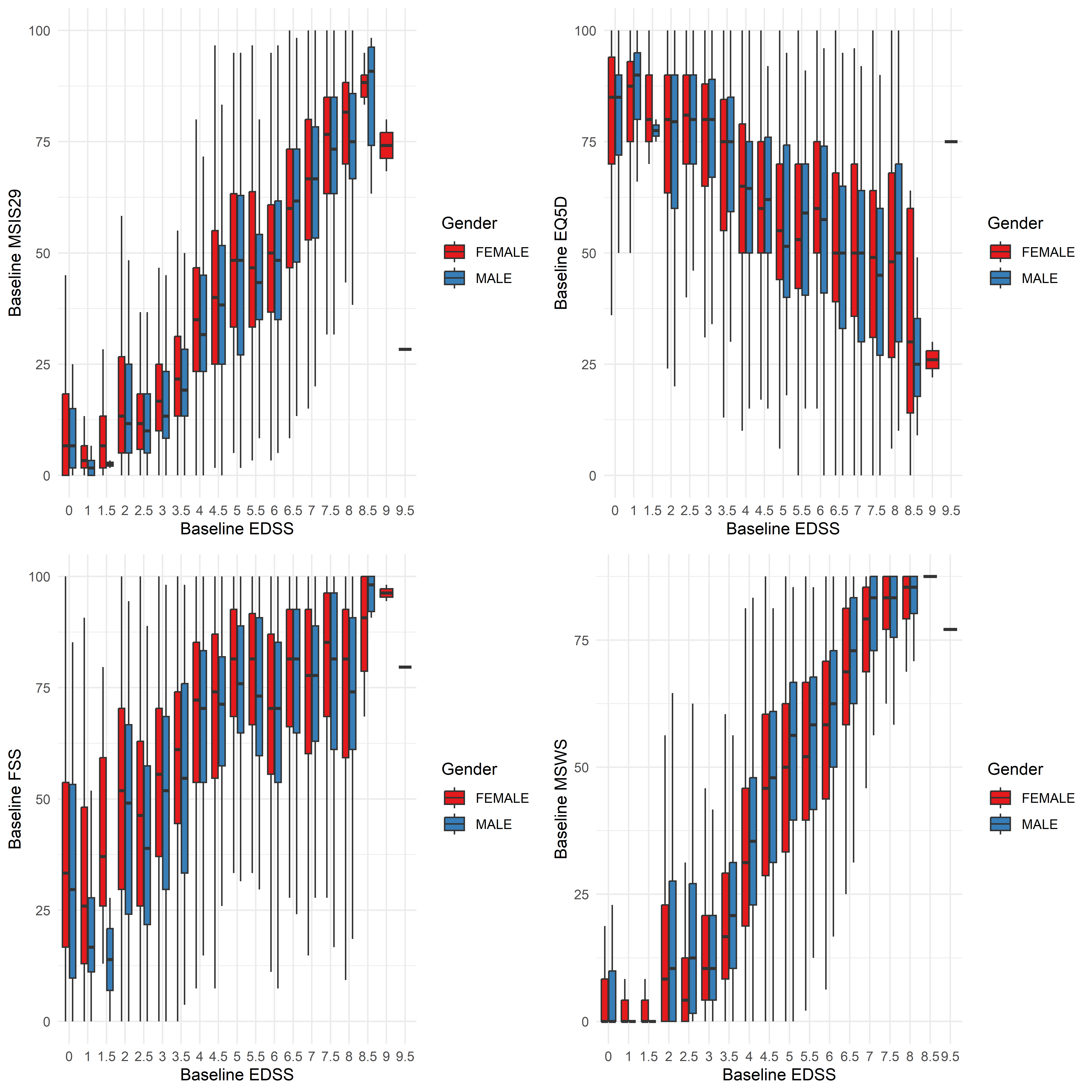
_

Supplementary figure 3: boxplots showing the relationship between EDSS and other severity measures, stratified by gender. The x axis shows the baseline EDSS. The y axes show the other MS severity measures. .

**Ethnicity and age of onset**

To determine whether year of diagnosis impacted on age of symptom onset and diagnosis, we conducted sensitivity analyses adjusting for year of diagnosis in addition to the covariates in the primary analysis (i.e. gender, progressive-onset disease, and ethnicity). The statistically-significant associations between South Asian and Black ethnicity and earlier onset/diagnosis persisted in this sensitivity analysis:

**
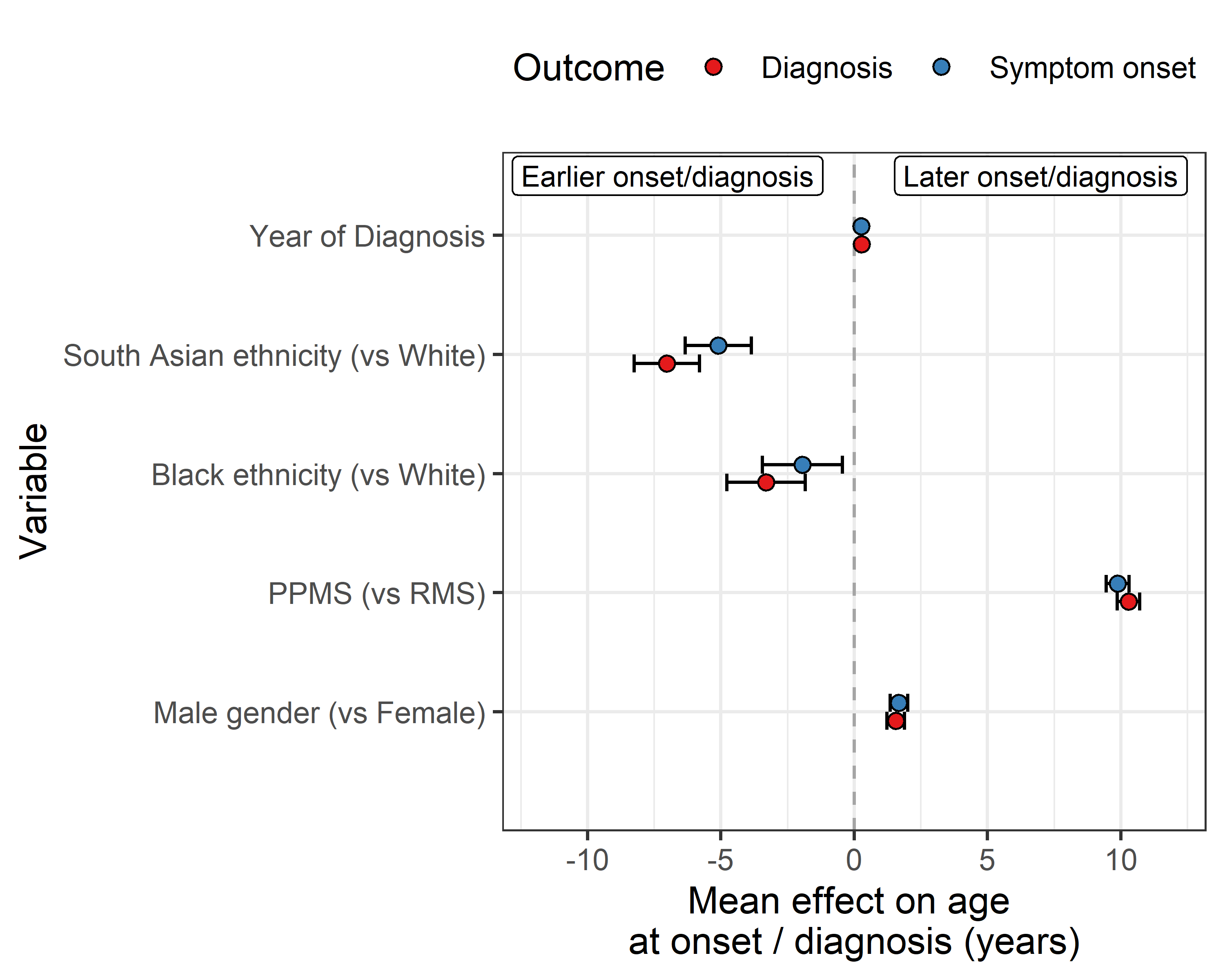
**

Supplementary figure 4: forest plot showing the estimated association between each covariate and age at symptom onset / diagnosis, with 95% confidence intervals. Blue points indicate the model for symptom onset, red points for age at diagnosis.

**Cross-sectional severity**

We ran a range of sensitivity analyses to confirm the lack of association between ethnicity and severity outcome measures. In a range of models adjusted for additional confounders (in addition to age at assessment, gender, and progressive-onset disease), we did not find strong evidence to support such a relationship. We observed weak evidence (Unadjusted *P* < 0.05) for lower walking-related disability (MSWS) in Black participants, and lower MSIS scores in South Asian participants, however neither of these associations withstood correction for multiple testing.

|  |  | Black | | South Asian | |
| --- | --- | --- | --- | --- | --- |
| Model | Outcome | Beta coefficient (95% CI) | P | Beta coefficient (95% CI) | P |
| Age at diagnosis | EDSS | 0.2 (-0.2 - 0.6) | 0.211 | -0.2 (-0.6 - 0.1) | 0.091 |
| Age at diagnosis | MSIS | -1.2 (-5.7 - 3.7) | 0.299 | 1.5 (-3 - 5.7) | 0.248 |
| Age at diagnosis | EQ5D | -1.7 (-5.3 - 2) | 0.183 | -0.5 (-3.7 - 2.8) | 0.384 |
| Age at diagnosis | FSS | -1.9 (-7.1 - 2.8) | 0.224 | -0.3 (-4.9 - 4.1) | 0.437 |
| Age at diagnosis | MSWS | -4.3 (-8.9 - 0.7) | 0.046 | -4.1 (-8.5 - 0.1) | 0.031 |
| DMT | EDSS | 0.1 (-0.3 - 0.5) | 0.369 | -0.2 (-0.5 - 0.2) | 0.186 |
| DMT | MSIS | -2.5 (-7.3 - 2.5) | 0.144 | 2.8 (-1.5 - 7.5) | 0.093 |
| DMT | EQ5D | -1.1 (-4.9 - 2.5) | 0.293 | -1.3 (-4.7 - 2.2) | 0.240 |
| DMT | FSS | -2.8 (-8 - 1.9) | 0.122 | 0.1 (-4.7 - 4.6) | 0.484 |
| DMT | MSWS | -5.3 (-10.3 - -0.3) | 0.017 | -2.6 (-6.8 - 2.1) | 0.125 |
| University education | EDSS | 0.1 (-0.3 - 0.5) | 0.355 | 0 (-0.3 - 0.4) | 0.400 |
| University education | MSIS | -1.2 (-6 - 3.8) | 0.316 | 4.5 (0 - 8.9) | 0.025 |
| University education | EQ5D | -2.3 (-6.4 - 1.7) | 0.124 | -2.3 (-5.8 - 1.3) | 0.105 |
| University education | FSS | -1.9 (-7.6 - 3.4) | 0.243 | 1.3 (-3.1 - 5.8) | 0.269 |
| University education | MSWS | -5.1 (-10.1 - -0.2) | 0.020 | -1.3 (-5.8 - 2.9) | 0.277 |
| Year of diagnosis | EDSS | 0.2 (-0.3 - 0.6) | 0.224 | -0.2 (-0.5 - 0.2) | 0.125 |
| Year of diagnosis | MSIS | -0.7 (-5.1 - 3.9) | 0.391 | 2.5 (-1.9 - 6.9) | 0.124 |
| Year of diagnosis | EQ5D | -2.1 (-5.9 - 1.6) | 0.131 | -1.2 (-4.6 - 2) | 0.237 |
| Year of diagnosis | FSS | -1.9 (-7.1 - 3.1) | 0.230 | -0.1 (-5.1 - 4.7) | 0.449 |
| Year of diagnosis | MSWS | -3.7 (-8.2 - 1.4) | 0.080 | -3.2 (-7.2 - 1) | 0.067 |
| Diagnostic lag | EDSS | 0.2 (-0.3 - 0.6) | 0.224 | -0.2 (-0.5 - 0.2) | 0.125 |
| Diagnostic lag | MSIS | -0.7 (-5.1 - 3.9) | 0.391 | 2.5 (-1.9 - 6.9) | 0.124 |
| Diagnostic lag | EQ5D | -2.1 (-5.9 - 1.6) | 0.131 | -1.2 (-4.6 - 2) | 0.237 |
| Diagnostic lag | FSS | -1.9 (-7.1 - 3.1) | 0.230 | -0.1 (-5.1 - 4.7) | 0.449 |
| Diagnostic lag | MSWS | -3.7 (-8.2 - 1.4) | 0.080 | -3.2 (-7.2 - 1) | 0.067 |
| First symptom | EDSS | 0.2 (-0.3 - 0.6) | 0.224 | -0.2 (-0.5 - 0.2) | 0.125 |
| First symptom | MSIS | -0.7 (-5.1 - 3.9) | 0.391 | 2.5 (-1.9 - 6.9) | 0.124 |
| First symptom | EQ5D | -2.1 (-5.9 - 1.6) | 0.131 | -1.2 (-4.6 - 2) | 0.237 |
| First symptom | FSS | -1.9 (-7.1 - 3.1) | 0.230 | -0.1 (-5.1 - 4.7) | 0.449 |
| First symptom | MSWS | -3.7 (-8.2 - 1.4) | 0.080 | -3.2 (-7.2 - 1) | 0.067 |
| Age at symptom onset | EDSS | 0.2 (-0.3 - 0.6) | 0.217 | -0.2 (-0.6 - 0.1) | 0.113 |
| Age at symptom onset | MSIS | -0.9 (-5.5 - 3.6) | 0.347 | 2.2 (-2.1 - 6.6) | 0.161 |
| Age at symptom onset | EQ5D | -1.9 (-5.7 - 1.7) | 0.160 | -0.9 (-4.2 - 2.3) | 0.293 |
| Age at symptom onset | FSS | -1.7 (-6.7 - 3.2) | 0.267 | -0.1 (-4.5 - 4.8) | 0.497 |
| Age at symptom onset | MSWS | -4.2 (-9.8 - 1) | 0.054 | -3.6 (-8 - 0.8) | 0.052 |
| Matched | EDSS | 0.2 (-0.3 - 0.6) | 0.217 | -0.2 (-0.6 - 0.1) | 0.113 |
| Matched | MSIS | -0.9 (-5.5 - 3.6) | 0.347 | 2.2 (-2.1 - 6.6) | 0.161 |
| Matched | EQ5D | -1.9 (-5.7 - 1.7) | 0.160 | -0.9 (-4.2 - 2.3) | 0.293 |
| Matched | FSS | -1.7 (-6.7 - 3.2) | 0.267 | -0.1 (-4.5 - 4.8) | 0.497 |
| Matched | MSWS | -4.2 (-9.8 - 1) | 0.054 | -3.6 (-8 - 0.8) | 0.052 |
| Primary analysis | EDSS | 0.1 (-0.3 - 0.6) | 0.267 | -0.2 (-0.6 - 0.2) | 0.150 |
| Primary analysis | MSIS | -1.7 (-6.5 - 3.1) | 0.236 | 2 (-2.5 - 6.2) | 0.182 |
| Primary analysis | EQ5D | -1.5 (-5.2 - 2.3) | 0.228 | -0.7 (-4 - 2.5) | 0.344 |
| Primary analysis | FSS | -2.2 (-7.5 - 2.8) | 0.201 | -0.2 (-4.7 - 4.5) | 0.494 |
| Primary analysis | MSWS | -4.8 (-9.7 - 0.5) | 0.033 | -3.5 (-7.7 - 1.3) | 0.061 |

Supplementary table 1: beta coefficients, 95% confidence intervals and P values for the association between ethnicity and cross-sectional severity across a range of sensitivity analysis models.

**Power calculations**

We performed empirical power calculations for the cross-sectional (baseline) analysis by generating simulated datasets of the same size as the ethnic groups in the study with non-missing MSIS data (White n = 12,588, Black n=114, South Asian n=156). We simulated a normal distribution of MSIS scores as a simplification, using the same standard deviation (26) as that observed in the whole population. For the White reference group we used the observed population mean of 40. We truncated these distributions such that randomly-generated scores outside of the 0-100 range were resampled, i.e. these were truncated normal distributions. We performed the same procedure for the non-reference group, altering the mean of the distribution, i.e. the true population difference between groups. We estimated power at alpha < 5% as the proportion of 1,000 bootstrap iterations in which the linear regression model Wald test P value term was <0.05.

For the survival analysis, we performed empirical power calculations by simulating survival times (i.e. time to a 10-point step change in MSIS score) using exponential distributions with a fixed hazard of 0.1 (i.e. 1 event per 10 person-years) for the reference (White) group, which closely resembled the observed data. We used sample sizes derived from the number of people in the longitudinal cohort with non-missing MSIS data (N=60 Black participants, 81 South Asian participants, and 7748 White participants). We adjusted the hazard for the non-reference group by multiplying the reference hazard by a range of hazard ratios, from 1 (no effect) to 2 (a doubling of the hazard). Over 1,000 bootstrap iterations, we performed unadjusted Cox regression and calculated empirical power as the proportion of iterations with a P value of < 0.05.

**Acknowledgements**

We would like to acknowledge the UK MS Register Research Group collaborators: Alasdair Coles, Jeremy Chataway, Martin Duddy, Hedley Emsley, Helen Ford, Leonora Fisniku, Ian Galea, Timothy Harrower, Jeremy Hobart, Huseyin Huseyin, Christopher M Kipps, Monica Marta, Gavin V McDonnell, Brendan McLean, Owen R Pearson, David Rog, Klaus Schmierer, Basil Sharrack, Agne Straukiene, David V Ford.

**Author contributions**

BMJ and RD contributed to the conception and design of the study; BMJ, PT and JW contributed to the acquisition and analysis of data; All authors contributed to drafting the text or preparing the figures.

**Potential conflicts of interest**

The authors have no relevant competing interests to declare.

**Funding**

BMJ is funded by an Medical Research Council (MRC) Clinical Research Training Fellowship (CRTF) jointly supported by the UK MS Society (BMJ; grant reference: MR/V028766/1). This work was carried out at the Centre for Preventive Neurology Unit at Queen Mary University of London, which is partly funded by Barts Charity. The UK MS Register is funded by the UK MS Society.

**References**

1. Leddy S, Hadavi S, McCarren A, Giovannoni G, Dobson R. Validating a novel web-based method to capture disease progression outcomes in multiple sclerosis. *J Neurol*. 2013;260(10):2505-2510. doi:10.1007/s00415-013-7004-1

2. Roxburgh RHSR, Seaman SR, Masterman T, et al. Multiple Sclerosis Severity Score: using disability and disease duration to rate disease severity. *Neurology*. 2005;64(7):1144-1151. doi:10.1212/01.WNL.0000156155.19270.F8

3. Manouchehrinia A, Westerlind H, Kingwell E, et al. Age Related Multiple Sclerosis Severity Score: Disability ranked by age. *Mult Scler*. 2017;23(14):1938-1946. doi:10.1177/1352458517690618

4. Hobart J, Lamping D, Fitzpatrick R, Riazi A, Thompson A. The Multiple Sclerosis Impact Scale (MSIS-29): a new patient-based outcome measure. *Brain*. 2001;124(Pt 5):962-973. doi:10.1093/brain/124.5.962

5. Hobart JC, Riazi A, Lamping DL, Fitzpatrick R, Thompson AJ. Measuring the impact of MS on walking ability: the 12-Item MS Walking Scale (MSWS-12). *Neurology*. 2003;60(1):31-36. doi:10.1212/wnl.60.1.31

6. Rodgers J, Friede T, Vonberg FW, et al. The impact of smoking cessation on multiple sclerosis disease progression. *Brain*. 2022;145(4):1368-1378. doi:10.1093/brain/awab385
